# Propanaminium *p*-toluene­sulfonate

**DOI:** 10.1107/S1600536812019435

**Published:** 2012-05-05

**Authors:** Yu Jin

**Affiliations:** a Ordered Matter Science Research Center, Southeast University, Nanjing 211189, People’s Republic of China

## Abstract

In the crystal structure of the title salt, C_3_H_10_N^+^·C_7_H_7_O_3_S^−^, N—H⋯O hydrogen bonds involving the ammonium groups of the cations and the sulfonate O atoms result in the formation of a three-dimensional network.

## Related literature

For general background to ferroelectric metal-organic frameworks, see: Zhang *et al.* (2009 ▶). For related structures, see: Helvenston *et al.* (2006 ▶); Collier *et al.* (2006 ▶); Koshima *et al.* (2001 ▶).
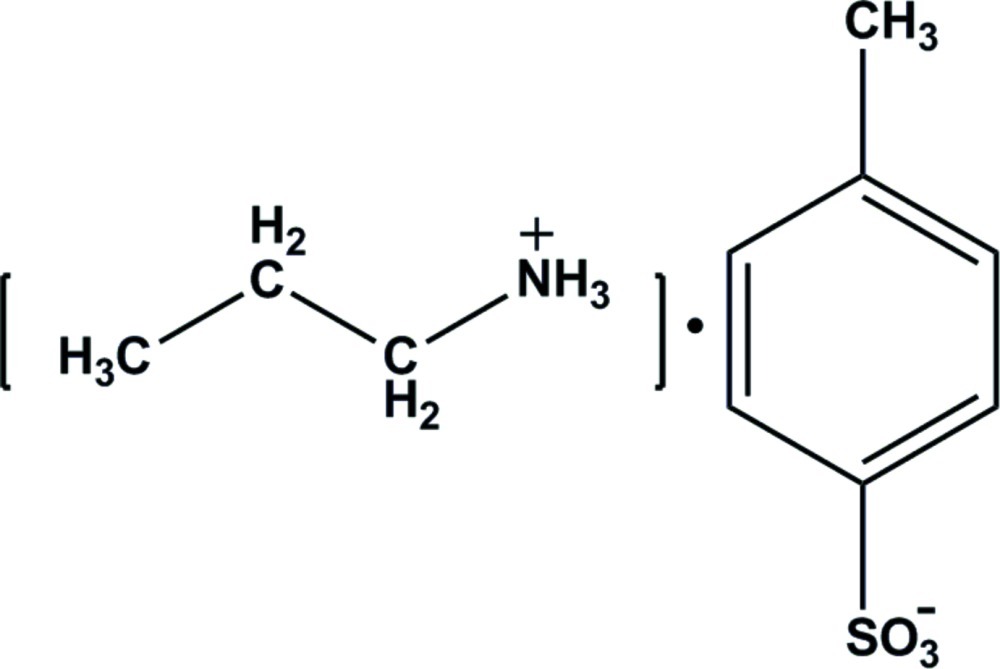


## Experimental

### 

#### Crystal data

C_3_H_10_N^+^·C_7_H_7_O_3_S^−^
*M* *_r_* = 231.31
Triclinic, 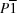

*a* = 5.6682 (11) Å
*b* = 7.3927 (15) Å
*c* = 13.817 (3) Å
α = 93.81 (3)°
β = 94.22 (3)°
γ = 91.27 (3)°
*V* = 575.9 (2) Å^3^
*Z* = 2
Mo *K*α radiation
μ = 0.27 mm^−1^
*T* = 293 K
0.30 × 0.30 × 0.20 mm

#### Data collection

Rigaku Mercury CCD diffractometer
Absorption correction: multi-scan (*CrystalClear*; Rigaku, 2005 ▶) *T*_min_ = 0.489, *T*_max_ = 1.000
6023 measured reflections
2639 independent reflections
1897 reflections with *I* > 2σ(*I*)
*R*_int_ = 0.040

#### Refinement

*R*[*F* ^2^ > 2σ(*F* ^2^)] = 0.069
*wR*(*F* ^2^) = 0.189
*S* = 1.03
2639 reflections
139 parameters
H-atom parameters constrained
Δρ_max_ = 1.02 e Å^−3^
Δρ_min_ = −0.52 e Å^−3^

### 

Data collection: *CrystalClear* (Rigaku, 2005 ▶); cell refinement: *CrystalClear*; data reduction: *CrystalClear*; program(s) used to solve structure: *SHELXS97* (Sheldrick, 2008 ▶); program(s) used to refine structure: *SHELXL97* (Sheldrick, 2008 ▶); molecular graphics: *SHELXTL* (Sheldrick, 2008 ▶); software used to prepare material for publication: *SHELXL97*.

## Supplementary Material

Crystal structure: contains datablock(s) I, global. DOI: 10.1107/S1600536812019435/im2372sup1.cif

Structure factors: contains datablock(s) I. DOI: 10.1107/S1600536812019435/im2372Isup2.hkl

Supplementary material file. DOI: 10.1107/S1600536812019435/im2372Isup3.cml

Additional supplementary materials:  crystallographic information; 3D view; checkCIF report

## Figures and Tables

**Table 1:** Hydrogen-bond geometry (Å, °)

*D*—H⋯*A*	*D*—H	H⋯*A*	*D*⋯*A*	*D*—H⋯*A*
N1—H1*A*⋯O2^i^	0.89	2.00	2.892 (4)	176
N1—H1*C*⋯O3^ii^	0.89	2.05	2.921 (4)	165
N1—H1*B*⋯O1	0.89	2.09	2.884 (4)	149

Symmetry codes: (i) 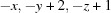; (ii) 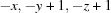.

**Fig. 1.**
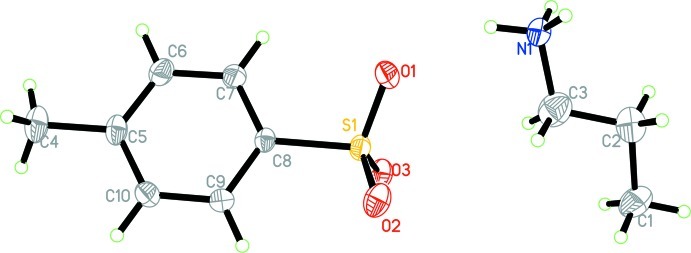
Molecular structure of the anion and cation of the title compound with displacement ellipsoids drawn at the 30% probability level.

**Fig. 2.**
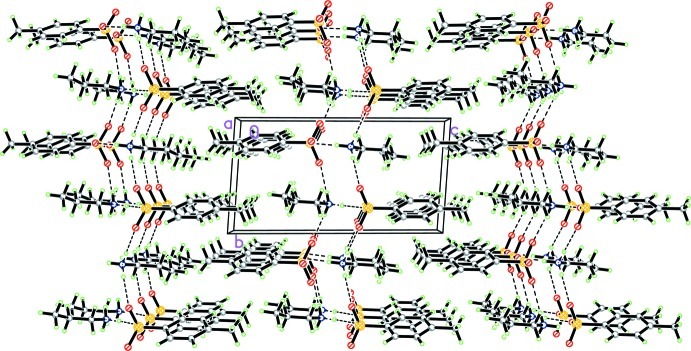
Crystal structure of the title compound viewed along the a axis. Intermolecular interactions are shown as dashed lines.

## supplementary crystallographic information

### Comment

Several crystal structures of *p*-toluenesulfonates have been reported previously, with the ammonium groups of the cations and the sulfonate O atoms efficiently establishing numerous hydrogen bond interactions (Helvenston *et al.*, 2006; Collier *et al.*, 2006; Koshima *et al.*, 2001). As an extension of this research, the synthesis and crystal structure of the title compound, (C_3_H_10_N^+^)(C_7_H_7_O_3_S^-^)^-^, aiming at enriching the series of *p*-toluenesulfonates is presented herein.

Ferroelectric compounds have a wide use in modern science. These compounds have displayed such technical applications as ferroelectric random access memories, ferroelectric field-effect transistors, piezoelectric sensors, nonlinear optical devices as a result of their excellent ferroelectric, piezoelectric, pyroelectric, and optical properties. Numerous new ferroelectric metal-organic coordination compounds corresponding to the necessary requirements for ferroelectric properties have been found, yet other necessary conditions, such as a phase transition, a good electric hysteresis loop and electric domain, and a dielectric anomaly, are often missed in these compounds (Zhang *et al.*, 2009). Therefore pure organic compounds have a tendency to make up for the drawbacks found in ferroelectric metal-organic coordination compounds. As part of our search for simple ferroelectric compounds, the title compound was investigated and its crystal structure is reported herein.

The asymmetric unit of the unit cell contains one anion and one cation that are shown in Fig. 1. Hydrogen bond interactions are listed in Table 1. The compound remains stable as a result of the existence of several hydrogen bond interactions formed in the crystal structure. These interactions tie the cations and anions together in a complex spatial geometry displayed in Fig2).

### Experimental

(C_3_H_10_N^+^)(C_7_H_7_O_3_S^-^) was formed from a mixture of propylamine, C_3_H_9_N (118.22 mg, 2.00 mmol), and *p*-toluenesulfonic acid, C_7_H_7_SO_3_H (172 mg, 1.00 mmol), and distilled water (10 mL). The reaction mixture was stirred a few minutes at room temperature, giving a clear solution. After evaporation of the solvent for a few days, block-shaped colorless crystals suitable for X-ray diffraction were obtained in 86% yield, filtered and washed with distilled water.

### Refinement

H atoms bound to carbon and nitrogen were placed at idealized positions [C—H = 0.93 to 0.97 Å and N—H = 0.89 Å] and allowed to ride on their parent atoms with *U*_iso_ fixed at 1.2 *U*_eq_(C,*N*).

### Crystal data

**Table:**

C_3_H_10_N^+^·C_7_H_7_O_3_S^−^	*Z* = 2
*M**_r_* = 231.31	*F*(000) = 248
Triclinic, *P*1	*D*_x_ = 1.334 Mg m^−^^3^
Hall symbol: -P 1	Mo *K*α radiation, λ = 0.71073 Å
*a* = 5.6682 (11) Å	Cell parameters from 3450 reflections
*b* = 7.3927 (15) Å	θ = 6.2–55.3°
*c* = 13.817 (3) Å	µ = 0.27 mm^−^^1^
α = 93.81 (3)°	*T* = 293 K
β = 94.22 (3)°	Block, colorless
γ = 91.27 (3)°	0.3 × 0.3 × 0.2 mm
*V* = 575.9 (2) Å^3^	

### Data collection

**Table:**

Rigaku Mercury CCD diffractometer	2639 independent reflections
Radiation source: fine-focus sealed tube	1897 reflections with *I* > 2σ(*I*)
Graphite monochromator	*R*_int_ = 0.040
ω scans	θ_max_ = 27.5°, θ_min_ = 3.0°
Absorption correction: multi-scan (*CrystalClear*; Rigaku, 2005)	*h* = −7→7
*T*_min_ = 0.489, *T*_max_ = 1.000	*k* = −9→9
6023 measured reflections	*l* = −17→17

### Refinement

**Table:**

Refinement on *F*^2^	Primary atom site location: structure-invariant direct methods
Least-squares matrix: full	Secondary atom site location: difference Fourier map
*R*[*F*^2^ > 2σ(*F*^2^)] = 0.069	Hydrogen site location: inferred from neighbouring sites
*wR*(*F*^2^) = 0.189	H-atom parameters constrained
*S* = 1.03	*w* = 1/[σ^2^(*F*_o_^2^) + (0.0894*P*)^2^ + 0.589*P*] where *P* = (*F*_o_^2^ + 2*F*_c_^2^)/3
2639 reflections	(Δ/σ)_max_ < 0.001
139 parameters	Δρ_max_ = 1.02 e Å^−^^3^
0 restraints	Δρ_min_ = −0.52 e Å^−^^3^

### Special details

**Table:**

Geometry. All e.s.d.'s (except the e.s.d. in the dihedral angle between two l.s. planes) are estimated using the full covariance matrix. The cell e.s.d.'s are taken into account individually in the estimation of e.s.d.'s in distances, angles and torsion angles; correlations between e.s.d.'s in cell parameters are only used when they are defined by crystal symmetry. An approximate (isotropic) treatment of cell e.s.d.'s is used for estimating e.s.d.'s involving l.s. planes.
Refinement. Refinement of *F*^2^ against ALL reflections. The weighted *R*-factor *wR* and goodness of fit *S* are based on *F*^2^, conventional *R*-factors *R* are based on *F*, with *F* set to zero for negative *F*^2^. The threshold expression of *F*^2^ > σ(*F*^2^) is used only for calculating *R*-factors(gt) *etc*. and is not relevant to the choice of reflections for refinement. *R*-factors based on *F*^2^ are statistically about twice as large as those based on *F*, and *R*- factors based on ALL data will be even larger.

### Fractional atomic coordinates and isotropic or equivalent isotropic displacement parameters (Å^2^)

**Table:**

	*x*	*y*	*z*	*U*_iso_*/*U*_eq_	
C1	0.1911 (8)	0.6760 (6)	0.2544 (3)	0.0653 (12)	
H1D	0.3047	0.7734	0.2710	0.098*	
H1E	0.1634	0.6599	0.1849	0.098*	
H1F	0.2509	0.5662	0.2790	0.098*	
C2	−0.0444 (8)	0.7220 (7)	0.2999 (3)	0.0667 (12)	
H2A	−0.1012	0.8366	0.2784	0.080*	
H2B	−0.1639	0.6284	0.2796	0.080*	
C3	−0.0004 (9)	0.7333 (7)	0.4042 (3)	0.0677 (12)	
H3A	0.1028	0.8379	0.4235	0.081*	
H3B	0.0829	0.6263	0.4229	0.081*	
C4	0.6719 (8)	0.7797 (6)	1.0672 (3)	0.0566 (10)	
H4A	0.8354	0.7483	1.0741	0.085*	
H4B	0.5784	0.6936	1.0980	0.085*	
H4C	0.6535	0.8988	1.0973	0.085*	
C5	0.5912 (6)	0.7771 (4)	0.9612 (2)	0.0390 (8)	
C6	0.3676 (6)	0.7134 (5)	0.9274 (2)	0.0426 (8)	
H6	0.2666	0.6691	0.9709	0.051*	
C7	0.2909 (6)	0.7140 (4)	0.8304 (2)	0.0379 (7)	
H7	0.1392	0.6712	0.8091	0.045*	
C8	0.4394 (5)	0.7782 (4)	0.7650 (2)	0.0287 (6)	
C9	0.6642 (6)	0.8422 (5)	0.7971 (2)	0.0403 (8)	
H9	0.7662	0.8846	0.7534	0.048*	
C10	0.7356 (6)	0.8427 (5)	0.8943 (2)	0.0444 (8)	
H10	0.8858	0.8886	0.9158	0.053*	
N1	−0.2160 (5)	0.7484 (4)	0.45840 (19)	0.0394 (7)	
H1A	−0.2958	0.8447	0.4408	0.059*	
H1B	−0.1749	0.7606	0.5220	0.059*	
H1C	−0.3069	0.6488	0.4450	0.059*	
O1	0.0916 (4)	0.7800 (4)	0.63485 (18)	0.0593 (8)	
O2	0.4559 (5)	0.9336 (3)	0.60426 (17)	0.0487 (6)	
O3	0.4344 (4)	0.6090 (3)	0.59619 (16)	0.0463 (6)	
S1	0.34710 (14)	0.77477 (11)	0.64010 (5)	0.0344 (3)	

### Atomic displacement parameters (Å^2^)

**Table:**

	*U*^11^	*U*^22^	*U*^33^	*U*^12^	*U*^13^	*U*^23^
C1	0.073 (3)	0.068 (3)	0.058 (3)	0.010 (2)	0.025 (2)	0.006 (2)
C2	0.072 (3)	0.070 (3)	0.057 (3)	0.004 (2)	−0.004 (2)	0.005 (2)
C3	0.079 (3)	0.065 (3)	0.059 (3)	−0.004 (2)	0.013 (2)	−0.003 (2)
C4	0.076 (3)	0.059 (2)	0.0333 (18)	0.015 (2)	−0.0078 (18)	0.0034 (17)
C5	0.050 (2)	0.0366 (17)	0.0294 (15)	0.0122 (15)	−0.0036 (14)	0.0001 (13)
C6	0.051 (2)	0.0438 (19)	0.0345 (16)	−0.0011 (16)	0.0067 (15)	0.0081 (14)
C7	0.0376 (17)	0.0409 (18)	0.0349 (16)	−0.0059 (14)	0.0036 (13)	0.0010 (13)
C8	0.0336 (15)	0.0271 (14)	0.0251 (13)	0.0081 (12)	0.0012 (11)	−0.0021 (11)
C9	0.0331 (17)	0.052 (2)	0.0361 (16)	−0.0024 (14)	0.0050 (14)	0.0024 (15)
C10	0.0329 (17)	0.058 (2)	0.0406 (18)	0.0017 (15)	−0.0019 (14)	−0.0033 (16)
N1	0.0468 (16)	0.0387 (15)	0.0326 (14)	0.0032 (12)	0.0017 (12)	0.0022 (12)
O1	0.0358 (14)	0.103 (2)	0.0379 (13)	0.0121 (14)	−0.0045 (11)	−0.0013 (14)
O2	0.0657 (17)	0.0429 (14)	0.0392 (13)	0.0082 (12)	0.0040 (12)	0.0136 (11)
O3	0.0599 (16)	0.0423 (13)	0.0356 (12)	0.0057 (11)	0.0045 (11)	−0.0088 (10)
S1	0.0370 (5)	0.0403 (5)	0.0255 (4)	0.0057 (3)	0.0007 (3)	0.0003 (3)

### Geometric parameters (Å, º)

**Table:**

C1—C2	1.552 (6)	C6—C7	1.378 (4)
C1—H1D	0.9600	C6—H6	0.9300
C1—H1E	0.9600	C7—C8	1.379 (4)
C1—H1F	0.9600	C7—H7	0.9300
C2—C3	1.442 (6)	C8—C9	1.381 (4)
C2—H2A	0.9700	C8—S1	1.764 (3)
C2—H2B	0.9700	C9—C10	1.374 (5)
C3—N1	1.482 (5)	C9—H9	0.9300
C3—H3A	0.9700	C10—H10	0.9300
C3—H3B	0.9700	N1—H1A	0.8900
C4—C5	1.500 (4)	N1—H1B	0.8900
C4—H4A	0.9600	N1—H1C	0.8900
C4—H4B	0.9600	O1—S1	1.446 (3)
C4—H4C	0.9600	O2—S1	1.447 (3)
C5—C6	1.380 (5)	O3—S1	1.445 (2)
C5—C10	1.383 (5)		
			
C2—C1—H1D	109.5	C7—C6—C5	121.3 (3)
C2—C1—H1E	109.5	C7—C6—H6	119.3
H1D—C1—H1E	109.5	C5—C6—H6	119.3
C2—C1—H1F	109.5	C6—C7—C8	120.0 (3)
H1D—C1—H1F	109.5	C6—C7—H7	120.0
H1E—C1—H1F	109.5	C8—C7—H7	120.0
C3—C2—C1	108.1 (4)	C7—C8—C9	119.8 (3)
C3—C2—H2A	110.1	C7—C8—S1	120.6 (2)
C1—C2—H2A	110.1	C9—C8—S1	119.6 (2)
C3—C2—H2B	110.1	C10—C9—C8	119.3 (3)
C1—C2—H2B	110.1	C10—C9—H9	120.4
H2A—C2—H2B	108.4	C8—C9—H9	120.4
C2—C3—N1	114.5 (4)	C9—C10—C5	122.1 (3)
C2—C3—H3A	108.6	C9—C10—H10	118.9
N1—C3—H3A	108.6	C5—C10—H10	118.9
C2—C3—H3B	108.6	C3—N1—H1A	109.5
N1—C3—H3B	108.6	C3—N1—H1B	109.5
H3A—C3—H3B	107.6	H1A—N1—H1B	109.5
C5—C4—H4A	109.5	C3—N1—H1C	109.5
C5—C4—H4B	109.5	H1A—N1—H1C	109.5
H4A—C4—H4B	109.5	H1B—N1—H1C	109.5
C5—C4—H4C	109.5	O3—S1—O1	113.29 (17)
H4A—C4—H4C	109.5	O3—S1—O2	111.81 (15)
H4B—C4—H4C	109.5	O1—S1—O2	112.99 (17)
C6—C5—C10	117.6 (3)	O3—S1—C8	106.01 (14)
C6—C5—C4	121.1 (3)	O1—S1—C8	105.90 (15)
C10—C5—C4	121.3 (3)	O2—S1—C8	106.13 (15)

### Hydrogen-bond geometry (Å, º)

**Table:**

*D*—H···*A*	*D*—H	H···*A*	*D*···*A*	*D*—H···*A*
N1—H1*A*···O2^i^	0.89	2.00	2.892 (4)	176
N1—H1*C*···O3^ii^	0.89	2.05	2.921 (4)	165
N1—H1*B*···O1	0.89	2.09	2.884 (4)	149

Symmetry codes: (i) −*x*, −*y*+2, −*z*+1; (ii) −*x*, −*y*+1, −*z*+1.
